# Supplementary figures and images for: Melanopsin Carboxy-terminus phosphorylation plasticity and bulk negative charge, not strict site specificity, achieves phototransduction deactivation
Source: PLoS One. 2020 Apr 1;15(4):e0228121. doi: 10.1371/journal.pone.0228121 (PMC7112210; doi:10.1371/journal.pone.0228121)

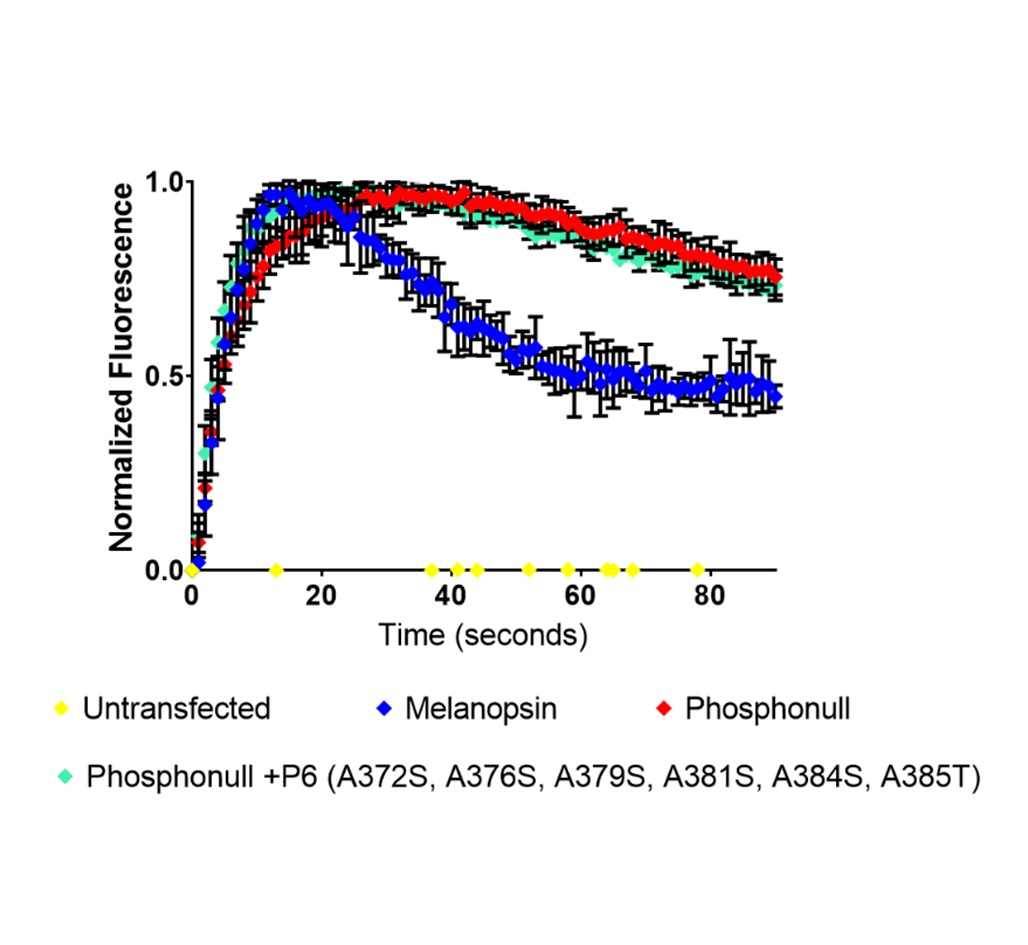

Supplement: S1 Fig — Calcium imaging of phopshonull + P6 (P-I sites mutated from alanine to serine and threonine residues). Error bars represent S.E.M. of three transfections. (TIF) [file pone.0228121.s001.tif]

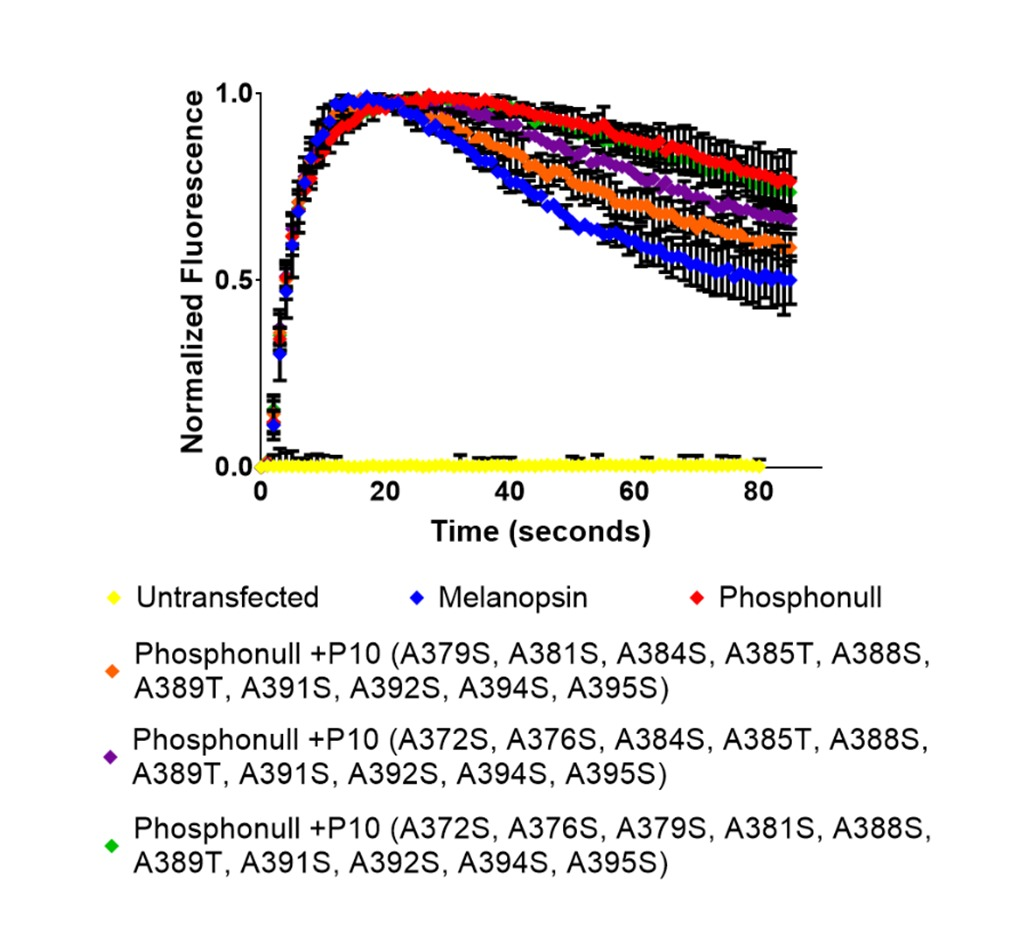

Supplement: S2 Fig — Calcium imaging of melanopsin phosphonull mutants with P-II mutated from alanine to serine and threonine residues and additional four sites on P-I mutated to serine and threonine residues. Mutant with P-II, and the four residues immediately before it mutated to serine and threonine residues (A379S, A381S, A384S, A385T) deactivates at the fastest rate among these mutants. Conversely, the mutant with P-II and the four additional residues furthest upstream on the C-terminus mutated to serine and threonine residues (A372S, A376S, A379S, A381S) displays the slowest deactivation rate, similar to phoshonull melanopsin. Error bars denote S.E.M. of three transfections. (TIF) [file pone.0228121.s002.tif]

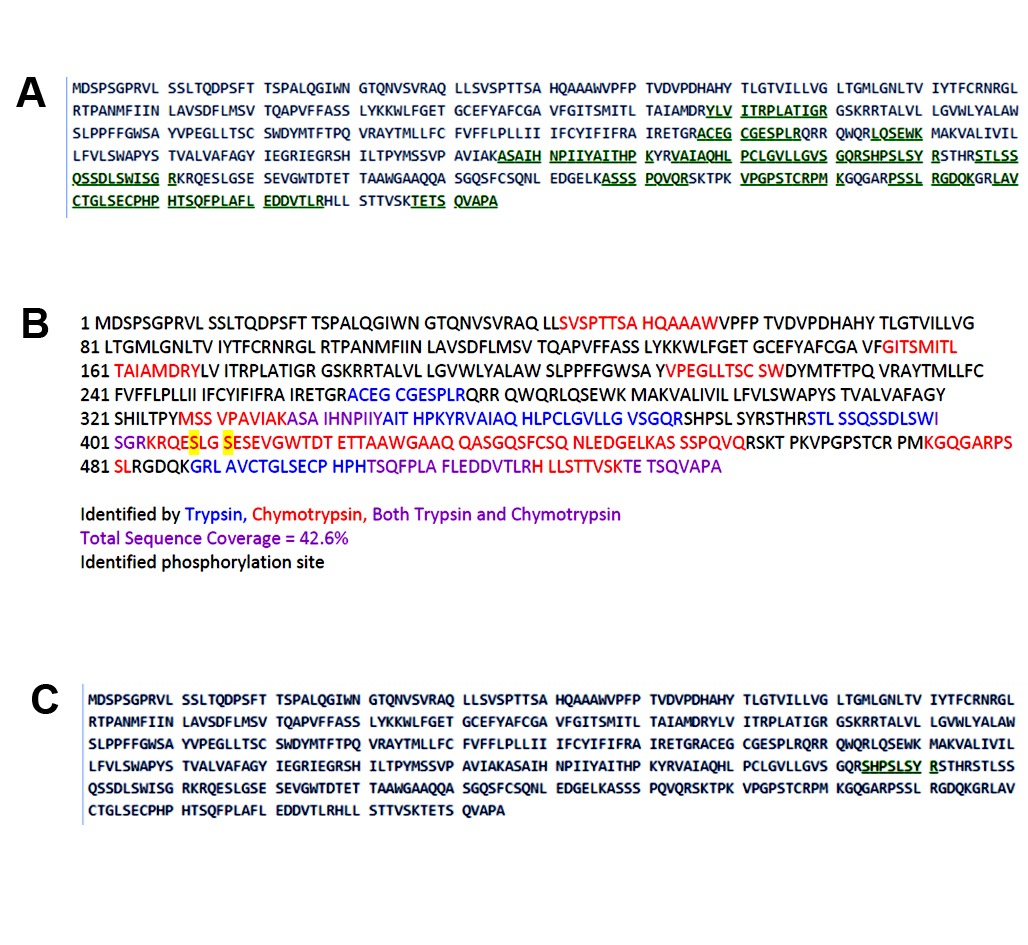

Supplement: S3 Fig — Melanopsin sequence coverage based on detected peptides following mass spectrometry denoted on its amino acid sequence. Sequence coverage following 1 min white light exposure (A), 30 min white light exposure (B), and after dark adaptation with no exposure to light (C). (TIF) [file pone.0228121.s003.tif]
